# Sensory Perception Quotient Reveals Visual, Scent and Touch Sensory Hypersensitivity in People With Fibromyalgia Syndrome

**DOI:** 10.3389/fpain.2022.926331

**Published:** 2022-07-05

**Authors:** Emma R. Dorris, James Maccarthy, Ken Simpson, Geraldine M. McCarthy

**Affiliations:** ^1^University College Dublin School of Medicine, University College Dublin, Dublin, Ireland; ^2^Northeast Fibromyalgia Support Group, Duleek, Ireland; ^3^Department of Rheumatology, Mater Misericordiae University Hospital, Dublin, Ireland

**Keywords:** fibromyalgia, hypersensitivity, hyposensitivity, chronic pain, public and patient involvement

## Abstract

**Background:**

Environmental sensitivity is commonly reported by people with fibromyalgia syndrome. People living with fibromyalgia syndrome frequently report hypersensitivity to noxious and non-noxious sensations. To date, there has been little empirical validation of sensory disturbance to non-noxious triggers. Environmental sensitivity is used as a diagnostic feature only in Bennet's alternative criteria for diagnosis of fibromyalgia, where it was ranked the second most important of the components for diagnosis, after number of pain sites. The aim of this study was to use a validated sensory measure to determine if people with fibromyalgia have greater sensory disturbances compared to people with other chronic pain conditions.

**Methods:**

This study used the Sensory Perception Quotient (SPQ) 92 question survey in adults with chronic pain conditions. A fibromyalgia group (*n* = 135) and a non-fibromyalgia chronic pain control group (*n* = 45) were recruited. All participants completed the SPQ as a self-report measure of sensory processing. In addition to the original SPQ scoring method, the Revised Scoring of the Sensory Perception Quotient (SPQ-RS) method was used to investigate self-reported hypersensitivity and hyposensitivity and the vision, hearing, taste, touch, and smell subscales. Chi-squared tests were used for categorical variables and Mann Whitney U, or Kruskal-Wallis H test were used to compare groups.

**Results:**

The fibromyalgia group reported significantly more sensitivity compared to the control group (*p* = 0.030). The fibromyalgia group reported significantly greater hypersensitivity (*p* = 0.038), but not more hyposensitivity (*p* = 0.723) compared to controls. The average fibromyalgia SPQ score (92.64 ± 23.33) was similar to that previously reported for adults with autism (92.95 ± 26.61). However, whereas adults with autism had broad range hypersensitivity, the fibromyalgia group reported significantly more hypersensitivity compared to the control group, but the range was restricted to vision (*p* = 0.033), smell (*p* = 0.049) and touch (0.040).

**Conclusions:**

These findings demonstrate greater sensory hypersensitivity in people with fibromyalgia compared to people with other chronic pain disorders. Greater hypersensitivity was restricted to touch, vision, and smell, all of which have previously been demonstrated to crosstalk with nociception.

## Introduction

Chronic pain is multifactorial by its very nature, with biological, psychological and social factors contributing to chronic pain syndromes (1). This complexity, in addition to the frequent lack of clinical biomarkers to aid diagnosis, means that chronic pain disorders can be difficult to understand, diagnose and treat. This leads to frustration for both health-care professionals and patients. Health care professionals may be uncertain of specific diagnoses, and people living with chronic pain conditions can be resentful that their symptoms are doubted (2, 3). Fibromyalgia syndrome (FMS) is a classic example of this, where despite significant evidence and recognition of it as a primary pain disorder, debate on its legitimacy was still common in the recent past (4, 5). This has left a legacy whereby people living with FMS report feelings of de-legitimization, betrayal, and anger toward the medical system (6, 7).

The recent development of nociplastic pain as a new pain descriptor provides validity for pain disorders such as FMS that were previously identified by stigmatizing terms (8). Nociplastic pain is now recognized although not entirely understood. It is used in the context of augmented central nervous system pain, augmented sensory processing, and altered pain modulation. Classic symptoms include widespread and/or amplified multifocal pain, fatigue, sleep, memory, and mood problems, as typified by FMS (2). The recognition of nociplastic pain, and increasing awareness of it in the medical vernacular, may help to prevent delays in diagnosis. However, differentiation between types of chronic pain conditions remains a challenge.

People living with FMS often report allodynia and pain hypersensitivity (9, 10). Environmental sensitivity is commonly reported by people with FMS (11, 12). Yet, environmental sensitivity is not used as a diagnostic feature in the American College of Rheumatology (ACR) 2011/2016 or the 2019 ACTTION-APS Pain Taxonomy (AAPT) fibromyalgia diagnosis criteria (13, 14). Although the AAPT criteria do not directly include it, it does refer to environmental sensitivity as a common feature of FMS that may be supportive of diagnosis. The Fibromyalgia Impact Questionnaire (FIQR) has a single question on environmental sensitivity, which was ranked second in importance as a diagnostic question in Bennet's alternative diagnosis criteria (15, 16).

The Patient Voice in Arthritis Research was established as an initiative to improve research relevance by collaborating with people living with rheumatic and musculoskeletal disease (RMD) in all aspects of research (17). Through this, people living with FMS in Ireland routinely expressed their frustration with a relative lack of research into FMS compared to other RMDs. In response, we co-designed a new research program directly with our FMS patient partners through a series of discussions and workshops. There were more than 200 people living with FMS involved in this process. Our FMS patient partners highlighted that environmental sensitivity had a large impact on their quality of life, particularly with regards to social impact.

There has been little empirical validation of sensory disturbance to non-noxious triggers in FMS to date. Given this gap in the evidence base and the impact of environmental sensitivity described by our FMS PPI partners, we investigated if people with FMS report greater sensory disturbances compared to people with other chronic pain conditions. The Sensory Perception Quotient (SPQ) is a validated measure for exploring self-reported hypersensitivity and hyposensitivity. Designed for adults, it specifically measures sensory sensitivity independent of reactive behaviors and complex cognitive processes (18). In this study we employ the SPQ to assess sensory sensitivity of people living with FMS compared to those living with other chronic pain conditions.

## Methods

### Aim

The aim of this study was to use a validated sensory measure to determine if people living with fibromyalgia have greater sensory disturbances compared to people living with other chronic pain conditions.

A secondary aim of the study was to investigate if specific sensory stimuli reported by people living with FMS were actively avoided more commonly in people living with FMS compared to those living with other chronic pain conditions.

### Public and Patient Involvement

People living with FMS were involved in the concept, research design, survey dissemination, identification of key points and writing and reviewing the manuscript and plain English summary. There were different PPI contributors involved at different stages, including 18 in the concept phase for this study (primary engagement channels: informal meeting, formal brainstorming workshop, informal communications), one on the selection committee and interview panel of the research associate working on this project, nine involved in the design phase [primary engagement channels: group meeting, closed Facebook group (as requested by PPI contributors)]. Three national FMS support charities disseminated the survey. Two people living with FMS were involved in identifying key points related to the study results (primary engagement channels: email and virtual meeting). One PPI contributor was involved with the review and revision of the manuscript, and with the drafting of the plain English summary. Additionally, the involvement of people living with FMS was critical for the focus on the potential impact of this study beyond the clinical and diagnostic setting.

### Participants

There were 289 participants recruited to the study. There were 266 female, 20 male and two identified as trans men. Of those recruited *n* = 15 did not meet the eligibility criteria. Participants that answered no (*n* = 9) or did not answer (*n* = 5) to the question on diagnosis of chronic pain disorders were excluded. Participants aged under 18 (*n* = 1) where excluded. All participants were recruited via an Irish chronic pain support charity: Chronic Pain Ireland, Arthritis Ireland, or FibroIreland. Approach was made via email, newsletter, or social media post containing a link to the survey. Participants self-declared their chronic pain condition (free text input). Participants were assigned to either an FMS (*n* = 200) or non-FMS (*n* = 74) chronic pain group.

### Measures

#### FMS-Sensory Triggers Questionnaire

Together with people living with FMS, a sensory triggers questionnaire was developed. It consisted of a single question with 35 item options. These options were derived directly from the project partners living with FMS (the NEFSG) who reported them as personal sensory stimuli. They also had significant input into the phrasing and format of the questionnaire. The questionnaire stated “Given the choice, what sensory stimuli do you/would you try to avoid in a public place? Tick all that apply” and gave the 35 stimuli options as tick box options.

#### Sensory Perception Quotient

The Sensory Perception Quotient (SPQ) is a validated 92-item adult self-report questionnaire measuring sensory sensitivity across modalities (18). SPQ is designed to investigate basic sensory processing and covers items for vision, hearing, touch, smell, and taste. It was designed for adults with autistic spectrum conditions and has been validated to measure sensory sensitivity in adults with and without autistic sensory conditions.

##### Power Calculation for the SPQ

Power analysis used data from the Tavassoli et al. study that first reported the SPQ measurement instrument (18). An alpha of 0.05, Beta 0.2 and Power 0.8 was used, with a 2:1 enrollment ratio. This produced a minimum total sample size of 99 (66 group 1: 33 group 2).

##### Scoring of the SPQ

The scoring of the SPQ was performed using the original scoring and the revised scoring as described (19).

Scoring for the full SPQ was performed as per (18). It includes all 92 questions, with a mix of forward and reverse scoring. A lower score indicates a higher sensory sensitivity. The full SPQ does not differentiate between hyper- and hypo-sensitivity.

Revised scored-SPQ (SPQ-RS) does not include the full 92 questions for scoring. It is a validated, revised scoring that differentiates between hyper- and hypo-sensitivity. It includes 79 of the SPQ items, of which 34 items correspond to the hypersensitivity scale, and 45 to the hyposensitivity scale. The scoring key used was as per (19). The SPQ-RS gives two scores: one for each scale, with a higher score indicating more atypical sensory sensitivity of the given type. Counter to the full SPQ, the SPQ-RS are coded such that higher scores indicate more atypical sensory sensitivity.

### Procedure

This study was exempt from full ethical review as data was collected anonymously. Low risk ethical exemption was approved by the University College Dublin Human Research Ethics Committee.

An electronic survey was used with three question sections. The survey was hosted on the SurveyMonkey Platform. Section one collected the inclusion/exclusion details. Section two was the FMS-sensory triggers questionnaire; and Section three was the 92-question SPQ. Section two used checkbox answers, with multiple selections accepted. Section three used a multiple-choice response with the 5-point Likert scale responses listed horizontally for each of the 92 items. Only one response per item was allowed. At the start of Section three the number of questions, estimated time to complete, and option to skip the section were detailed.

There was no time limit to complete the survey. There was no incentive offered to participants. The overall survey design and clarity was informed and reviewed by the PPI project partners.

Survey responses were exported to Excel. An IP check was performed to identify possible duplicate entries. None were identified. Responses underwent review for inclusion/exclusion criteria. Data was coded and imported into SPSS version 24. Pearson's Chi-squared test was used to test the distribution of categorical variables between groups. Mann-Whitney U tests were used to compare SPQ and SPQ-RS scores between groups. A *p*-value of <0.05 was considered statistically significant.

## Results

### Homogeneity Between FMS and Non-FMS Cohorts

No sex statistical differences were observed between groups (Chi-Square Statistic 1.328, *p* = 0.249). There was no observed statistical difference in co-morbidities between groups (Supplementary Table 1). The most common comorbidities were arthritis (*p* = 0.207), migraine (*p* = 0.590), irritable bowel syndrome (IBS, *p* = 0.178), hypothyroidism (*p* = 0.978), depression (*p* = 0.566), and anxiety (*p* = 0.849).

### Overall Sensory Sensitivity

All 92 questions must be completed for full SPQ scoring. There was *n* = 129 in the FMS group and *n* = 45 in the non-FMS group. The Mann Whitney U tests demonstrated a significant difference in the SPQ score between groups (*p* = 0.030). The FMS group had an average SPQ of 92.64 compared to 101.40 in the non-FMS group (Table 1), indicating that people living with FMS have greater overall sensory sensitivity. The SPQ has five subscales measuring vision, hearing, taste, smell, and touch. A statistically significant difference between groups was observed in the smell (*p* = 0.013) and touch (*p* = 0.007) subscales only (Table 1).

**Table 1:** Comparison of overall sensory sensitivity between FMS and non-FMS chronic pain groups.

	**FMS**	**Non-FMS**	**Significance** **(2-tailed)**
**SPQ score**			
Median	93.00	99.00	**0.030**
Mean	92.64	101.40	
SEM	2.054	2.720	
95% CI	88.57–96.70	95.92–106.88	
**Vision subscale**			
Median	25.00	27.00	0.173
Mean	25.16	26.47	
SEM	0.457	0.717	
95% CI	24.25–26.06	25.02–27.91	
**Hearing subscale**			
Median	25.00	27.00	0.217
Mean	25.05	26.04	
SEM	0.490	0.777	
95% CI	24.09–26.02	24.48–27.61	
**Taste subscale**			
Median	14.00	14.00	0.352
Mean	14.27	15.09	
SEM	0.410	0.665	
95% CI	13.46–15.08	13.75–16.43	
**Smell subscale**			
Median	14.00	17.00	**0.013**
Mean	14.09	16.84	
SEM	0.637	0.975	
95% CI	12.83–15.35	14.88–18.81	
**Touch subscale**			
Median	13.00	16.00	**0.007**
Mean	14.06	16.96	
SEM	0.566	0.839	
95% CI	12.94–15.18	15.26–18.65	

*Statistically significant values (p < 0.05) are in bold*.

#### Hypersensitivity Sensory Domains

The SPQ-RS uses 79 of the 92 questions. There was *n* = 135 in the FMS group and *n* = 45 in the non-FMS group. Mann Whitney U tests demonstrated a significant difference in the hypersensitivity score between FMS and non-FMS groups (*p* = 0.038). People living with FMS reported being more hypersensitive to sensory stimuli compared to the non-FMS group (Table 2). In the five subdomains, the FMS group had significantly greater hypersensitivity compared to the non-FMS group in the vision (*p* = 0.033), smell (*p* = 0.049), and touch (*p* = 0.040) subdomains (Table 2).

**Table 2:** Comparison of hypersensitivity between FMS and non-FMS chronic pain groups.

	**FMS**	**Non-FMS**	**Significance (2-tailed)**
**Hypersensitivity score SPQ-RS**			
Median	31.00	27.00	**0.038**
Mean	31.36	27.38	
SEM	0.934	1.308	
95% CI	29.52–33.21	24.74–30.01	
**Vision subscale**			
Median	9.00	7.00	**0.033**
Mean	8.46	7.22	
SEM	0.268	0.426	
95% CI	7.93–8.46	6.36–8.08	
**Hearing subscale**			
Median	6.00	5.00	0.142
Mean	6.39	5.58	
SEM	0.268	0.449	
95% CI	5.86–6.92	4.67–6.48	
**Taste subscale**			
Median	5.00	5.00	0.72
Mean	4.90	4.73	
SEM	0.201	0.315	
95% CI	4.51–5.30	4.10–5.37	
**Smell subscale**			
Median	6.00	5.00	**0.049**
Mean	6.21	5.18	
SEM	0.275	0.405	
95% CI	5.66–6.75	4.36–5.99	
**Touch subscale**			
Median	5.00	5.00	**0.040**
Mean	5.40	4.67	
SEM	0.186	0.266	
95% CI	5.03–5.77	4.13–5.20	

*Statistically significant values (p < 0.05) are in bold*.

#### Hyposensitivity Subdomain

There was *n* = 135 in the FMS group and *n* = 45 in the non-FMS group. Mann Whitney U tests showed no significant difference in hyposensitivity between groups (*p* = 0.723). There was no statistically significant difference between groups on any of the five sub-domains (Table 3).

**Table 3:** Comparison of hyposensitivity between FMS and non-FMS chronic pain groups.

	**FMS**	**Non-FMS**	**Significance (2-tailed)**
**Hyposensitivity RS-SPQ**			
Median	11.00	10.00	0.723
Mean	11.98	11.84	
SEM	0.586	0.826	
95% CI	10.82–13.14	10.18–13.51	
**Vision subscale**			
Median	1.00	1.00	0.318
Mean	1.81	1.49	
SEM	0.161	0.255	
95% CI	1.49–2.13	0.97–2.00	
**Hearing**			
Median	3.00	3.00	0.202
Mean	3.24	2.80	
SEM	0.161	0.249	
95% CI	2.93–3.56	2.30–3.30	
**Taste**			
Median	2.00	2.00	0.991
Mean	2.27	2.13	
SEM	0.166	0.219	
95% CI	1.94–2.60	1.69–2.58	
**Smell**			
Median	1.00	1.00	0.238
Mean	1.75	2.20	
SEM	0.221	0.404	
95% CI	1.31–2.19	1.39–3.01	
**Touch**			
Median	2.00	3.00	0.391
Mean	2.91	3.22	
SEM	0.154	0.327	
95% CI	2.61–3.22	2.56–3.88	

### FMS-Sensory Triggers

A larger proportion of the cohort completed the FMS-triggers questionnaire. There was *n* = 200 in the FMS group and *n* = 74 in the non-FMS group. Of the 35 environmental trigger items, there was a significance statistical difference between cohorts for ten of the items (Table 4). The FMS group reported avoiding the following social environmental items more frequently compared to the non-FMS group: bright light (*p* = 0.036), artificial light (*p* = 0.024), glare (*p* = 0.005), flashing light (*p* < 0.001), food odors (*p* = 0.001), auditory mechanical background noise (*p* = 0.035), multiple screens on display (*p* = 0.021), fluctuating temperature (*p* = 0.034) and bright décor (*p* = 0.023). The non-FMS group reported being more likely to avoid soft seating compared to the FMS group (*p* = 0.002).

**Table 4:** Frequency table comparing sensory triggers in the social environment between FMS and non-FMS chronic pain groups.

**Given the choice, what sensory stimuli do you/would you try to avoid in a public place?**	**FMS** ***n* (%)**	**Non-FMS** ***n* (%)**	***P*-value**
Bright light	138 (69.0)	41 (55.4)	**0.036**
Low light	8 (4.0)	1 (1.4)	0.275
Sunlight	52 (26.0)	12 (16.2)	0.089
Artificial light	75 (37.5)	17 (23.0)	**0.024**
Glare	132 (66.0)	35 (47.3)	**0.005**
Flashing light	144 (72.0)	36 (48.6)	**<0.001**
Soft seating	10 (5.0)	12 (16.2)	**0.002**
Vinyl seating	38 (19.0)	9 (12.2)	0.182
Fabric seating	4 (2.0)	4 (5.4)	0.137
Course seating	56 (28.0)	20 (27.0)	0.873
Food odors/smells	84 (42.0)	15 (20.3)	**0.001**
Outdoors odors/smells	40 (20.0)	9 (12.2)	0.133
Perfume aromatherapy	72 (36.0)	24 (32.4)	0.583
Coffee odor/smell	20 (10.0)	4 (5.4)	0.232
Alcohol odor/smell	42 (21.0)	13 (17.6)	0.529
Mechanical background noise	107 (53.5)	29 (39.2)	**0.035**
Conversational (talking) background noise	84 (42.0)	23 (31.1)	0.100
Loud music	127 (63.5)	44 (59.5)	0.540
Any music	16 (8.0)	3 (4.1)	0.254
All background noise	50 (25.0)	16 (21.6)	0.561
High technology usage	57 (28.5)	19 (25.7)	0.643
Single screen on display	18 (9.0)	3 (4.1)	0.172
Multiple screens on display	96 (48.0)	24 (32.4)	**0.021**
High temperature	97 (48.5)	30 (40.5)	0.241
Low temperature	87 (43.5)	30 (40.5)	0.660
Fluctuating temperature	99 (49.5)	26 (35.1)	**0.034**
Wind (for example open/closing door)	90 (45.0)	30 (40.5)	0.509
Seating close together	110 (55.5)	37 (50.0)	0.461
Narrow aisles	98 (49.0)	37 (50.0)	0.883
Wide aisles	0 (0.0)	0 (0.0)	.
Bright décor	53 (26.5)	10 (13.5)	**0.023**
Dull décor	11 (5.5)	2 (2.7)	0.334
Flamboyant décor	41 (20.5)	13 (17.5)	0.588
Obtrusive décor	37 (18.5)	9 (12.2)	0.213

*Statistically significant values (p < 0.05) are in bold*.

## Discussion

In this study, we have demonstrated that people living with FMS report greater sensory dysfunction compared to people living with other chronic pain conditions. Vision, smell and touch subdomains are significantly different between FMS and non-FMS chronic pain patients. Of note, the vision subscale was not statistically significant using the overall (standard) SPQ sensitivity scoring, but it was significant using the revised SPQ-RS hypersensitivity scoring. This may be due to the difference is the questions included in the SPQ and SPQ-RS. The revised method, SPQ-RS, removed thirteen questions as they were considered poor measures due to being either indirectly associated with sensory sensitivity or textually ambiguous (19). The vision subscale for the overall sensitivity scoring had twenty questions, the revised SPQ-RS hypersensitivity vision subscale had ten questions and the SPQ-RS hyposensitivity vision subscale had seven questions. These revisions may explain why vision was statistically significant for hypersensitivity, but not the overall sensitivity quotient.

Increased sensitivity to noxious stimuli in fibromyalgia has been attributed to central sensitization of dorsal horn neurons (20). Recently, Goebel et al. uncovered that IgG autoantibodies may be the mechanism by which hypersensitivity to noxious stimuli occur in FMS (21). People living with FMS report that their hypersensitivity is not limited to painful stimuli, but rather can include a variety of non-noxious stimuli found in the everyday environment, such as light.

Atypical sensory processing is not included within the ACR diagnostic criterion of FMS (13, 22). Despite widespread reporting of hypersensitivity to non-noxious triggers by people living with FMS, environmental sensitivity is only used as a diagnostic feature in the alternative criteria by Bennett et al. (16), where it was ranked the second most important component for FMS diagnosis, after number of pain sites. There is an increasing movement toward person-centered assessment of chronic pain conditions (23). This is due, in part, to the recognition that health care professionals consistently underestimate pain compared to patients (24). Self-reported pain, therefore, is now the gold-standard (25). Any additional elements for clinical diagnosis must by practical for use. Here, we demonstrate that the validated self-assessed SPQ questionnaire, specifically the SPQ-RS for hypersensitivity, may have application in the diagnosis of FMS. As it is self-administered, it could be easily adapted to use within a clinical setting. Our study provides evidence for future research into the utility of the SPQ-RS hypersensitivity subdomain alongside the other continuous quantitative measures scored under the current 2016 ACR diagnostic survey for fibromyalgia (13).

Social isolation is a common in people living with FMS (26). The reasons underpinning this isolation are complex, and include perceived invalidation and lack of social support (27). The lack of objective markers of FMS has been a persistent problem in FMS diagnosis, management and research (28, 29). This lack of objective pathologic evidence in fibromyalgia historically resulted in the dismissal of fibromyalgia as a psychological problem, with a resultant ineffective management and understanding of the condition (30). However, an additional element of social isolation may be hypersensitivity to non-noxious triggers in social environments. We have demonstrated that people with FMS report hypersensitivity to non-noxious triggers. Additionally, we have identified specific environmental triggers that people with FMS are more likely to actively avoid. The identification of visual hypersensitivity on the SPQ-RS scale and the higher frequency of people living with FMS who would actively avoid bright, artificial, flashing lights or glare, indicate a high aversion to visual triggers.

Accessible applications, such as Access Earth (31) already exist to help those with physical disabilities plan and navigate their environments and social activities. The identification of specific environmental triggers that contribute to social isolation in those with sensory disabilities may help to forward the inclusion of sensory triggers in the rating of community, business, and social environment accessibility. The inclusion of sensory accessibility information in these formats could empower people with sensory disturbances, including those living with FMS, to make informed choices based on clear, publicly available sensory accessibility information about the local environment. Thus, instead of avoiding social interactions due to fear of the cognitive fatigue that can be induced by sensory triggers in the environment, people living with FMS would be empowered to make choices of appropriate, accessible environments that minimize their sensory load.

## Conclusions

People living with FMS report greater hypersensitivity to non-noxious sensory stimuli compared to people living with other chronic pain conditions. Sensory overload can have significant impact on quality of life. Recognition of sensory disability and adjustments or adaptations to make the environment more accessible to those with sensory disabilities may assist in alleviating some of the social isolation experienced by people living with FMS.

## Data Availability Statement

The raw data supporting the conclusions of this article will be made available by the authors, without undue reservation.

## Ethics Statement

The studies involving human participants were reviewed and approved by UCD Human Research Ethics Committee. Written informed consent for participation was not required for this study in accordance with the national legislation and the institutional requirements.

## Author Contributions

ED and GM: conception. ED, KS, JM, and GM: design of the work, interpretation of data, and substantively revised the manuscript. ED and JM: acquisition and analysis of data. ED: drafted the work. All authors contributed to the article and approved the submitted version.

## Funding

This study was funded by the Irish Society of Rheumatology Patient Improvement Fund.

## Conflict of Interest

The authors declare that the research was conducted in the absence of any commercial or financial relationships that could be construed as a potential conflict of interest.

## Publisher's Note

All claims expressed in this article are solely those of the authors and do not necessarily represent those of their affiliated organizations, or those of the publisher, the editors and the reviewers. Any product that may be evaluated in this article, or claim that may be made by its manufacturer, is not guaranteed or endorsed by the publisher.

## Abbreviations

ACR: American College of Rheumatology
AAPT, Analgesic, Anesthetic: and Addiction Clinical Trial Translations Innovations Opportunities and Networks (ACTTION)- American Pain Society (APS) Pain Taxonomy
FMS: Fibromyalgia syndrome
IBS: Irritable bowel syndrome
NEFSG: Northeast fibromyalgia support group
PPI: Public and Patient Involvement
SPQ: Sensory Perception Quotient
SPQ-RS: Sensory Perception Quotient-Revised Scoring.
