# Supplementary material for: Sensory Perception Quotient Reveals Visual, Scent and Touch Sensory Hypersensitivity in People With Fibromyalgia Syndrome
Source: Front Pain Res (Lausanne). 2022 Jul 5;3:926331. doi: 10.3389/fpain.2022.926331 (PMC9294149; doi:10.3389/fpain.2022.926331)
Supplement: Supplementary file 1 [file Table_1.pdf]

Supplementary Material

Sensory Perception Quotient reveals visual, scent and touch sensory hypersensitivity in people with fibromyalgia syndrome

Emma R Dorris, James Maccarthy, Ken Simpson, Geraldine M McCarthy

1 Supplementary Tables

Supplementary Table 1: Comorbidities between groups

	**FMS** n (%)	**Non-FMS** n (%)	**Chi Statistic**	**P-value** (2-sided)
**Arthritis**	29 (22.3)	14 (31.8)	1.589	0.207
**Migraine**	19 (14.6)	5 (11.4)	0.291	0.590
**IBS**	15 (11.5)	2 (4.5)	1.813	0.178
**Hypothyroidism**	12 (9.2)	4 (9.1)	0.001	0.978
**Depression**	11 (8.5)	5 (11.4)	0.330	0.566
**Anxiety**	10 (7.7)	3 (6.8)	0.036	0.849

**Lupus**	5 (3.8)	1 (2.3)	0.243	0.622
**PCOS**	6 (4.6)	2 (4.5)	0.000	0.985
**Diabetes**	4 (3.1)	2 (4.5)	0.212	0.645
**Endometriosis**	4 (3.1)	3 (6.8)	1.185	0.276
